# Effective Prevention and Management Tools for Metabolic Syndrome Based on Digital Health-Based Lifestyle Interventions Using Healthcare Devices

**DOI:** 10.3390/diagnostics12071730

**Published:** 2022-07-16

**Authors:** Jung-Hun Lee, Kang-Hyun Lee, Hee-Jin Kim, Hyun Youk, Hee-Young Lee

**Affiliations:** Department of Emergency Medicine, Yonsei University Wonju College of Medicine, Wonju 26426, Korea; leejhuns@yonsei.ac.kr (J.-H.L.); dhheejin@yonsei.ac.kr (H.-J.K.); yhmentor@gmail.com (H.Y.); hylee3971@yonsei.ac.kr (H.-Y.L.)

**Keywords:** metabolic syndrome, healthcare, digital-health-based lifestyle intervention, health information management

## Abstract

Digital health-based lifestyle interventions (e.g., mobile applications, short messaging service, wearable devices, social media, and interactive websites) are widely used to manage metabolic syndrome (MetS). This study aimed to confirm the utility of self-care for prevention or management of MetS. We recruited 106 participants with one or more MetS risk factors from December 2019 to September 2020. Participants were provided five healthcare devices and applications. Characteristics were compared at baseline and follow-up to examine changes in risk factors, engagement, persistence, and physical activity (analyzed through device use frequency and lifestyle interventions performed). Participants with 1–2 MetS risk factors showed statistically significant reductions in waist circumference (WC) and blood pressure (BP). Participants with ≥3 MetS risk factors showed statistically significant reductions in risk factors including weight, body mass index, WC, BP, and fasting blood sugar (FBS). The prevention and improvement groups used more healthcare devices than the other groups. Smartwatch was the most frequently used device (5 times/week), and physical activity logged more than 7000 steps/week. WC, BP, and FBS of the improvement group were reduced by more than 40%. Based on engagement, persistence, and physical activity, digital health-based lifestyle interventions could be helpful for MetS prevention and management.

## 1. Introduction

### 1.1. Background

Metabolic syndrome (MetS) is a growing global public health challenge [1,2]. Several population studies have highlighted the increased prevalence of MetS [3,4,5,6]. The National Health and Nutrition Examination Survey (2017–2018) has reported the prevalence of MetS as approximately 38.3% [F5]; the corresponding values reported by the Korean National Health and Nutrition Survey in 2017 were 28.1% and 18.7%, respectively [6]. The increase in MetS was associated with several factors that were the result of changing lifestyles, primarily aging, eating habits, physical inactivity, sedentary work, long working hours, and stress [7,8]. MetS encompasses factors such as abdominal fat, hypertension, dyslipidemia, and glucose intolerance. In addition, it is a risk factor for type 2 diabetes, coronary heart disease, and other cardiovascular diseases [2,9,10,11,12]. Pharmacological interventions may help delay or manage complications associated with MetS. The prime emphasis in the management of the MetS is to mitigate the modifiable, underlying risk factors (obesity, physical inactivity, and an atherogenic diet) through lifestyle changes [1,12,13,14,15,16,17]. Several previous studies have examined the impact of digital health technologies on lifestyle changes and health outcomes [12,13,14,15,16,17,18,19]. Digital health is defined as information communication technology that supports health through electronic and mobile health solutions and using big data, computational genomics, and artificial intelligence [20]. Digital health may improve population health by increasing access to medical services and uptake of interventions [20,21,22,23,24]. Mobile applications, short messaging service (SMS), wearable devices, social media, and interactive websites have been used as intervention methods [14,16,17,25,26,27].

### 1.2. Lifestyle Interventions and Self-Care

Previous studies have reported on weight loss in participants with MetS and obesity using remote monitoring and healthcare services combined with conventional treatment [14,16,17,25,26,28]. Other studies examined the impact of lifestyle interventions delivered by health coaches alongside activity monitoring [27]. These studies showed activity changes among the participants based on feedback received from the monitoring service. However, these studies only reported the effects of healthcare services based on digital health-based lifestyle interventions. In addition, no detailed study aiming to show that lifestyle interventions and self-care are required to improve MetS has been conducted. Lifestyle interventions need to be conducted according to the different patient characteristics, and self-care should be encouraged to prevent and manage MetS. Digital health-based lifestyle interventions may be key to achieving lifestyle changes and improving health [22,23,29]. People need to be able to adhere to lifestyle behaviors in order to make consistent and long-lasting changes. The importance of adherence in the treatment of obesity or diabetes related to MetS has been widely described [30,31]. The effectiveness of lifestyle intervention depends on timely confirmation that self-care is being maintained. In cases where it is not being maintained, digital health coordinators can provide personalized feedback and support. Lifestyle changes may be assessed using direct and indirect measures, including the levels of engagement, persistence, and physical activity, all of which contribute to intervention uptake, helping improve MetS outcomes.

### 1.3. Objective

This study aimed to confirm the utility of self-care prevention and management tools using healthcare devices. We analyzed the effectiveness of digital health-based lifestyle interventions, which involved the use of healthcare devices to support lifestyle changes and sustained self-care. The participants with pre-MetS and MetS were divided into groups defined by the changes to the number of risk factors observed; the differences in the levels of engagement, persistence, and physical activity were examined.

## 2. Materials and Methods

### 2.1. Definition of Metabolic Syndrome

The diagnostic criteria of the National Cholesterol Education Program: Adult Treatment Panel III and the International Diabetes Federation were used in this study [2,18]. Abdominal obesity was defined based on the Korean Society for the Study of Obesity waist circumference (WC) cut-off values, which were used to determine MetS in this population [6]. MetS was defined as the presence of three or more of the following: (1) WC of ≥90 cm in men or ≥85 cm in women; (2) fasting blood sugar (FBS) levels of ≥100 mg/dL; (3) systolic/diastolic blood pressure (SBP/DBP) of ≥130/85 mmHg; (4) high-density lipoprotein cholesterol (HDL-C) levels of <40 mg/dL in men or <50 mg/dL in women; and (5) triglyceride (TG) levels of ≥150 mg/dL. In this study, participants with pre-MetS (defined as having 1 or 2 risk factors) were included for prevention of MetS.

### 2.2. Design

The study participants were enrolled from December 2019 to September 2020 using participants from the Wonju cohort study. The Wonju cohort study included a community-based cohort in Wonju, Korea, and medical examinations and epidemiological investigations were performed to identify cardiovascular and chronic diseases [32]. From the Wonju cohort study, participants with one or more MetS risk factors were invited to confirm the study explanation and intention to participate. The criteria for participation in this study were 40–80 years of age, one or more MetS risk factors, and no difficulty in using healthcare devices. Participants were excluded if they were taking medication, had difficulty using healthcare devices, or could not follow instructions related to the intervention. The participants were evaluated at two time points. The first assessment examined risk factors for MetS. Selected participants received five self-care healthcare devices. Lifelog data were collected by an application installed on the participants’ smartphone. After 6 weeks, a lifestyle intervention was introduced to induce lifestyle changes and continuous engagement with the healthcare devices. Follow-up assessments of risk factors for MetS were performed at 26 weeks. This study protocol was approved by the Institutional Review Board of the Wonju College of Medicine, Yonsei University (CR319089). The study protocol was registered at the Clinical Research Information Service (KCT0005783).

### 2.3. Participants

**Figure 1:**
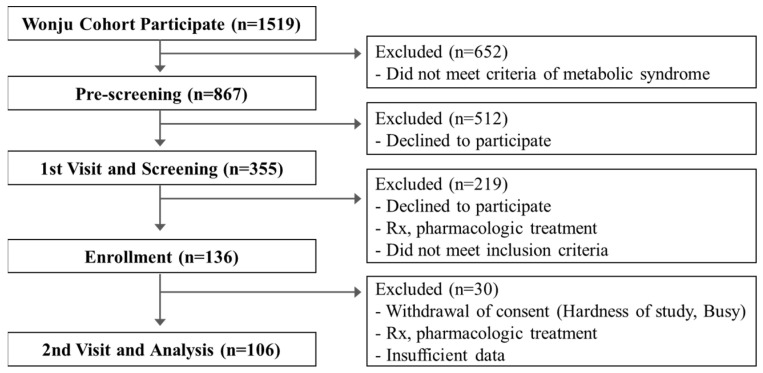
Flowchart of the study population selection.

The Wonju cohort study included 1519 participants, of whom 867 participants had one or more MetS risk factors. Participants were recruited to the clinical trial via phone calls, and 355 participants confirmed their participation. The participants were assessed during a hospital visit, and study aims were explained. Among them, a total of 136 participants presented with more than one risk factor and agreed to participate in the clinical trial. Each participant received five healthcare devices. Finally, data from a total of 106 participants examined at the 26-week follow-up assessment were included in the analysis. One participant taking diabetes mellitus medication, twenty-two participants who withdrew their consent (due to difficulties participating in the study or unavailability, among others), and seven participants who did not use the provided devices for more than half the study period were excluded from the analysis (Figure 1).

### 2.4. Collection of Lifelog Data Using Healthcare Devices

Lifelog data are the personal health data gathered daily and automatically by the provided application/healthcare devices. The provided devices were a blood pressure monitor (Omron HEM-9200T, Omron Healthcare, Japan); self-monitoring blood glucose (SMBG) (CareSens N Premier, I-SENS, Korea); weight scale (Efilscale, LifeSemantics, Korea); smart tape measure (PIE, Bagel Labs, Korea); and smartwatch (Galaxy Watch Active 1, Samsung Electronics, Suwon, Korea). These devices were connected to an application, which recorded all the relevant measurements and automatically transferred the lifelog data from the participants’ smartphones to a web-based server. It was recommended that the devices be used three or more times per week. Medical staff had access to the lifelog data in real time through a designated website.

### 2.5. Group Classification

Participants who received lifestyle interventions were included in the analysis. The participants with pre-MetS/MetS were divided into two groups, according to the baseline and follow-up (26 weeks) assessment findings. The participants with pre-MetS with the aim of prevention were divided according to changes in their risk factors: the prevention group, with reduced and consistent risk factors, and the non-prevention group, with increased risk factors. The participants with the MetS with the aim of management were divided into the improvement group, with reduced risk factors, and the non-improvement group, with consistent and increased risk factors.

### 2.6. Engagement, Persistence, and Physical Activity

Engagement was defined as the frequency of device use per week, based on pre-specified criteria that involved two rules: (1) all five healthcare devices were used more than the minimum frequency required; (2) total weekly frequency of device use was greater than the minimum frequency required. “Persistence” referred to the continuous number of satisfied engagements. The maximum persistence during the study period was analyzed. Physical activity was measured with a step counter, which was embedded in the provided smartwatch. In this study, the prevention and improvement groups were used as a reference for lifestyle changes. Digital health-based lifestyle interventions were implemented during weeks 6, 8, and 10. Text messages were delivered from week 6, phone calls were made from week 8, and face-to-face re-training was conducted from week 10. Any questions from the participants were resolved by the medical staff via phone calls or visits.

### 2.7. Prevention and Management of Metabolic Syndrome

The characteristics of all study groups were analyzed on the basis of the risk factors examined at both the baseline and follow-up. The number of risk factors and levels of engagement, persistence, and physical activity were confirmed.

### 2.8. Statistical Analysis

The participants’ characteristics at baseline were compared with those at follow-up using the paired *t*-test. The data are presented as counts, percentages, means, and standard deviations. An independent samples *t*-test was used to compare differences between the two groups. Analyses were performed using IBM SPSS Statistics 25 (SAS Institute, Cary, NC, USA). *p*-values of <0.05 were considered statistically significant.

## 3. Results

### 3.1. Participant’s Characteristics between the Baseline and Follow-Up

The participants’ characteristics are presented in Table 1. Significant differences in WC, SBP, DBP, and HDL-C were observed in the pre-MetS participants. The average number of risk factors increased from 1.7 (0.5) to 1.6 (1.1) (*p* > 0.05). Significant differences in MetS were observed in weight, BMI, risk factors, WC, SBP, DBP, and FBS. The average number of risk factors decreased from 3.6 (0.6) to 2.4 (1.1) (*p* < 0.05). There were 42 participants in the pre-MetS prevention group with reduced and consistent risk factors and 14 in the non-prevention group with increased risk factors. The MetS in the improvement group with reduced risk factors comprised 43 participants and the non-improvement group with consistent and increased risk factors 17 participants.

### 3.2. Effectiveness of Digital Health-Based Lifestyle Interventions

**Figure 2:**
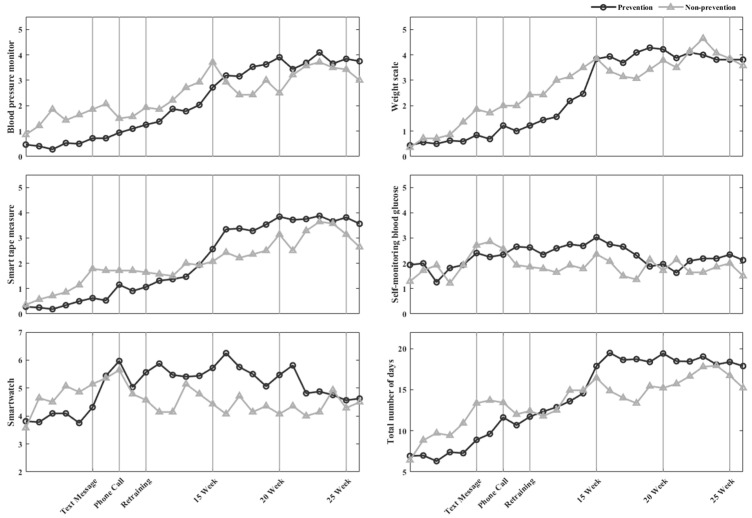
In pre-MetS, the number of days healthcare devices were used per week.

**Figure 3:**
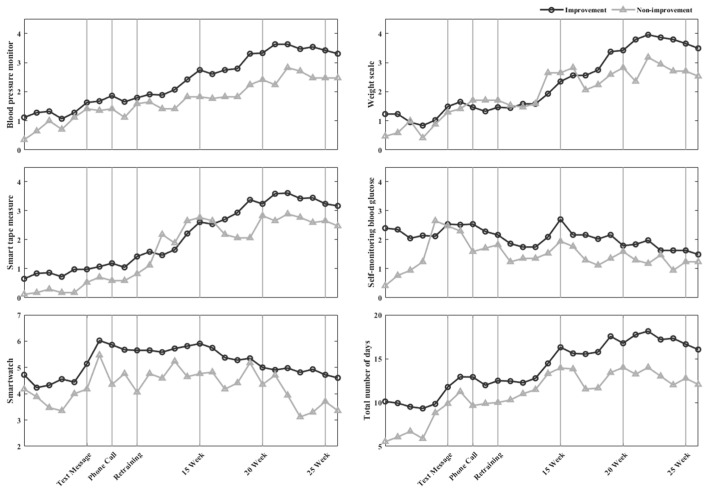
In MetS, the number of days healthcare devices were used per week.

The frequency of device use is presented in Figure 2 and Figure 3. The frequency of healthcare device use increased when lifestyle interventions were implemented; specifically, during week 10, when re-training was provided, the frequency of device use was greater than those in weeks 6 and 8 when text messages and phone calls were introduced, respectively. In pre-MetS/MetS, the level of engagement with blood pressure monitors, scales, and smart tape measures increased gradually and stabilized from week 14 onwards.

### 3.3. Characteristic of Self-Care Using Healthcare Devices

#### 3.3.1. The Frequency of Healthcare Device Use

The frequencies of healthcare device use are presented in Table 2. For those with pre-MetS, there were significant differences in the use of a smart tape measure, SMBG, smartwatch, and total number of use days between the prevention and non-prevention groups (*p* < 0.05). For those with the MetS, there were significant differences between the improvement and non-improvement group (*p* < 0.05) in the use of the five healthcare devices and the total number of use days.

#### 3.3.2. Engagement and Persistence

We focused on changes in the prevention and improvement groups after week 14 and determined the criteria for the achievement of engagement and persistence (Table 2). The rules of engagement were defined as follows. Rule 1: Weight scale, smart tape measure, and blood pressure monitor were used on more than 3 days per week; the SMBG was used on more than 2 days per week, with the pre- and post-meal use counted as a single event. Smartwatch was used on more than 5 days per week. Rule 2: The total number of days when devices were used was more than 16. Weekly engagement criteria were satisfied when either of the rules was met. The overall engagement levels in the prevention and non-prevention groups of the pre-MetS participants during the 26-week period were 10.6 (6.1) and 9.9 (6.3), respectively (*p* > 0.05), and in the improvement and non-improvement groups of the pre-MetS participants 10.2 (5.8) and 7.1 (6.1), respectively (*p* < 0.05). The maximum levels of persistence in the prevention, non-prevention, improvement, and non-improvement groups were 8.9 (5.7), 6.8 (4.7), 7.5 (4.8), and 5.4 (4.9), respectively. Although the engagement and persistence levels in the prevention and improvement groups were relatively high, the differences were not significant (*p* > 0.05).

#### 3.3.3. Physical Activity

Changes to physical activity levels were statistically significant after 14 weeks (Table 2). The average numbers of steps walked per day were 7162.4 (4714.0), 5837.1 (5034.0), 7444.3 (5337.3), and 4878.6 (4543.1) in the prevention, non-prevention, improvement, and non-improvement groups, respectively (*p* < 0.05). In the prevention and improvement groups with high physical activity there were relatively large decreases in the WC and BP.

### 3.4. Prevention and Management of Metabolic Syndrome

Changes in risk factors between baseline and follow-up are presented (Table 2). For the pre-MetS participants, while the prevention and non-prevention groups showed no statistical differences in the baseline, significant differences in the follow-up were observed. Furthermore, the two groups had the greatest WC and BP values at baseline. The prevention group showed large decreases in WC (19%) and BP (53%), whereas the non-prevention group showed large increases in the HDL-C (50%) and TG (43%). For the participants with the MetS, the two groups had a higher number of all risk factors. The improvement group showed significant reductions in the WC (44%), BP (49%), FBS (40%), HDL-C (19%), and TG (19%), whereas the non-improvement group showed no significant changes.

## 4. Discussion

We presented digital health-based lifestyle interventions for the prevention and management of MetS, which involved the use of healthcare devices to support lifestyle changes and sustained self-care. Previous studies compared the outcomes of participants who received lifestyle interventions and those who did not. Some evidence suggests that lifestyle interventions may be effective. However, this study examined the participants’ characteristics based on the changes to risk factors and proposed a tool to prevent and manage MetS using self-care healthcare devices. We quantified and confirmed the frequencies of use of healthcare devices for continuous interest and self-care and recommended the appropriate frequency of use. Differences among groups were observed despite the use of the same lifestyle interventions in all the participants. These findings suggest that some modifications to lifestyle interventions may be required based on the participant characteristics in order to increase the likelihood of lifestyle changes.

### 4.1. Management Tool of MetS Using Healthcare Devices

We provided participants with five healthcare devices: a blood pressure monitor, weight scale, SMBG, smart tape measure, and smartwatch. Lifelog data were used to evaluate the levels of engagement, persistence, and physical activity. The level of engagement was represented by the number of times a device was used per week by the participants to maintain self-care easily. Self-care utilizes values measured through healthcare devices, but it can take a long time to confirm meaningful changes in the measured values. In the short term, it may be difficult to recognize the changed amount because the value measured by the healthcare device is a small or similar change. This make it difficult to motivate participants, as it is difficult for them to identify health improvements. In addition, the values may have been affected by the participants’ body positions, measurement sites, and device measurement errors [33]. The number of steps walked reflected the levels of physical activity directly and was easy for the participants to check; therefore, the frequencies of use and step counts were the measures of lifestyle change in this study.

### 4.2. Digital Health-Based Lifestyle Intervention

Previous findings have suggested that digital health-based lifestyle interventions are powerful ways to improve health. Research related to the management of MetS is changing from the classic intervention methods such as web-based education program, e-mail feedback, telephone, and SMS to an intervention method using application, wearable devices, and coaching through health monitoring [14,16,17,25,26,27]. In this study, phone calls, text messages, and re-training were applied from week 6 to help increase the levels of engagement with lifestyle interventions. The impact of text messages and phone calls was low. Face-to-face re-training helped increase engagement. Re-training focused on problems with smartphone use, connection with healthcare devices, and device use. The uptake of self-care in this context may be limited by the user’s ability to interact with a device; these limitations should be addressed in a timely manner.

Oh et al. provided a body composition monitor (including weight measurement) and a pedometer to the intervention group (*n* = 212) and conducted health counseling through the recorded data. The control group (*n* = 210) was provided with a weight scale and a step counter for 24 weeks. Participants were recommended to measure at least three times a week. In the test group and control group, weight decreased by 2.2 (3.6) kg and 0.8 (2.8) kg, respectively, and the decrease was higher in the intervention group that performed healthcare [26]. Jae-Min Park et al. provided smartwatches for participants with and without MetS. Participants (*n* = 43) with MetS had statistically significant decreases in BMI, WC, TG, and BP. Participants (*n* = 68) without MetS showed statistically significant differences in BMI, WC, and HDL-C [17]. Mao et al. provided participants (*n* = 763) with a scale, pedometer, and blood pressure monitor and performed health coaching through live video, phone calls, and text messages through an application. During the 4-month study, an average of 3.2% of total weight was lost, and 28.6% of participants decreased their weight by more than 5% [27]. Huh et al. provided wearable devices to participants (*n* = 20) and asked them to walk regularly for 12 weeks. Participants were provided with self-feedback on their exercise volume through a mobile application. After 12 weeks, risk factors decreased from 3.4 to 2.9. There was a decrease in 11 (55%) participants and no change in 7 (35%) participants. Physical activity was 7510.04 (3525) steps [25].

In this study, the risk factors of participants with pre-MetS decreased from 1.7 (0.5) to 1.6 (1.1) (*p* > 0.05), 18 (39%) participants decreased, 14 (30%) maintained, and 14 (30%) increased. Weight was 0.25 (1.62) kg, and 26 (57%) participants decreased risk factors (*p* > 0.05). The risk factors of participants with MetS decreased from 3.6 (0.6) to 2.4 (1.1) (*p* < 0.01), 43 (72%) participants decreased, 16 (27%) maintained, and 1 (1%) increased risk factors. Weight was 0.89 (2.13) kg, and 40 (67%) participants decreased (*p* < 0.01). In the prevention and improvement groups, physical activity was 7162.4 (4714.0), and 7444.3 (5337.3) steps, and in the non-prevention and non-improvement groups, it was as low as 5837.1 (5033.0), and 4878.6 (4543.1) steps. Compared with the previous study, the improvement of risk factors was high. Although weight loss and physical activity were low, a large number of participants did demonstrate weight loss. This is thought to be the result of the intervention provided to the participants focusing on self-care rather than weight management.

### 4.3. Engagement, Persistence, and Physical Activity

The prime emphasis in the management of MetS is the mitigation of the modifiable, underlying risk factors (obesity, physical inactivity, and atherogenic diet) through lifestyle changes. Increased levels of physical activity help reduce weight and body mass index, improving the overall risk factor profile. Engagement levels were assessed using those in prevention and improvement groups as references, as these groups effectively managed the risk factors for pre-MetS and MetS. Two rules defined engagement criteria: the number of device use days per week (rule 1) and the total number of device use days (rule 2). These rules helped prevent engagement failures due to problems with the healthcare devices. Persistence was measured as devices use at least three times a week, as based on existing studies [26]. However, it was considered that there would be differences depending on the convenience of the participants in using the equipment [34], so the criteria were referred to the prevention and improvement groups. In fact, the smart watch that can be worn on the wrist had the highest use at five times a week, and the scale, smart tape measure, and blood pressure monitor were measured more than three times a week. On the other hand, in the case of a blood glucose meter that requires body blood taken through a lancet, the frequency of use at twice a week was low. Engagement was defined by these characteristics, and persistence was reflected as the amount of continuously satisfied engagement. In addition, we hypothesized that lifestyle changes may be associated with increased persistence. In this study, engagement and persistence levels in the prevention and improvement groups were relatively high but not significant. Therefore, we had a relatively short period of time remaining after 14 weeks of stable use of the healthcare devices.

### 4.4. Change in Risk Factors

The prevention and improvement groups used more of the provided healthcare devices than the other groups. The levels of satisfied engagement and maximum persistence were also high. The prevention group showed large decreases in the WC (19%) and BP (53%), whereas the non-prevention group showed large increases in the HDL-C (50%) and TG (43%). The improvement group showed significant reductions in the WC (44%), BP (49%), FBS (40%), HDL-C (19%), and TG (19%), whereas the non-improvement group showed no significant changes. The prevention and improvement groups had the highest levels of engagement, persistence, and physical activity with a significant decrease in the number of risk factors. The number of risk factors observed in the non-prevention group of pre-MetS participants increased. Despite moderate levels of physical activity, the WC and BP increased slightly, and the HDL-C and TG levels increased significantly, suggesting challenges related to dietary habits. Dietary habits are associated with hyperglycemia, hypertriglyceridemia, hypertension, low HDL cholesterol levels, and abdominal obesity [35,36]. In the non-improvement group of the MetS participants, the five risk factors did not change or changed slightly. This group had lower engagement, persistence, and physical activity levels than the other groups. Continuous self-care monitoring using lifestyle interventions is recommended.

### 4.5. Drop-Out

The drop-out rate in a previous study was approximately 48% in the first 4 weeks [25] and 20% during the observation period [26]. Dropout risk is associated with participant age, lack of familiarity with smartphones or wearable devices, and use of mobile application. In contrast, in this study, 23 of 138 (16%) participants dropped out. Reasons for dropping out included difficulties associated with study participation, being busy, and unwillingness to continue. All participants had experience with long-term cohort studies and understood clinical trial and healthcare service protocols, both of which may account for the lower drop-out rate in this study than that in previous studies. However, 7 (6%) participants failed to use the provided devices for >13 weeks.

### 4.6. Limitations

There are several limitations to this study. First, we performed a digital health-based intervention in a single group. Additionally, in some studies, even if equipment for self-care management is provided, the improvement effect of MetS may be insufficient if motivation is not achieved through appropriate intervention [37,38]. For self-care through the proposed level of engagement, persistence, and physical activity, it is important to inform participants about steady health management by using the application, phone, etc., consistently [30,31]. The participants’ experience using wearable devices was not considered; engagement levels may be affected by device familiarity. The age of the participants in this study is also high, which might make it difficult to use healthcare devices, and participants may require help [38]. If you are not familiar with the connection between the healthcare devices and the application or the use of the devices, data omissions or errors may occur. Improper data collection may affect analysis results. This study accounted only for the presence of a risk factor rather than for when it occurred. Future studies should examine the impact of risk factor timing on disease onset. The number of people included in the classified group was low. A detailed protocol for the proposed lifestyle interventions should be developed and validated in clinical trials.

## 5. Conclusions

We presented digital health-based lifestyle interventions as prevention and management tools for MetS, which involved the use of healthcare devices to support lifestyle changes and sustained self-care. In addition, we quantified and confirmed the frequencies of use of healthcare devices for continuous interest and self-care and suggested the appropriate frequencies of use. The prevention and improvement group used more of the provided healthcare devices than the other groups. The use of digital health-based lifestyle interventions may increase engagement and persistence in self-healthcare.

## Figures and Tables

**Table 1:** Participant characteristics at baseline and follow-up assessments.

Characteristics	Pre-MetS (*n* = 46)			MetS (*n* = 60)		
	Baseline	Follow-Up	*p*-Value	Baseline	Follow-Up	*p*-Value
General Characteristics, mean (SD)						
Sex (male/female) (*n*)	17/29	-	-	25/35		
Age (years)	63.9 (6.8)	-	-	65.2 (5.9)		
Height (cm)	160.1 (8.0)	159.9 (8.1)	0.11	160.0 (9.3)	160.0 (9.2)	0.93
Weight (kg)	62.1 (9.1)	61.8 (8.9)	0.29	67.9 (11.4)	67.1 (11.3)	<0.01
BMI (kg/m^2^)	24.2 (2.9)	24.2 (2.9)	0.75	26.4 (2.7)	26.0 (2.7)	<0.01
Risk factors	1.7 (0.5)	1.6 (1.1)	0.57	3.6 (0.6)	2.4 (1.1)	<0.01
Risk factors of metabolic syndromes, mean (SD)						
WC (cm)	87.9 (7.5)	84.4 (7.2)	<0.01	93.3 (7.1)	88.7 (8.4)	<0.01
SBP (mmHg)	138.0 (15.7)	125.5 (15.2)	<0.01	140.1 (15.7)	127.0 (13.0)	<0.01
DBP (mmHg)	90.1 (9.1)	79.9 (9.2)	<0.01	89.6 (8.1)	80.3 (9.0)	<0.01
FBS (mg/dL)	92.4 (9.2)	91.5 (10.9)	0.56	101.7 (10.8)	96.9 (9.1)	<0.01
HDL-C (mg/dL)	55.1 (9.0)	52.9 (10.0)	<0.05	46.6 (9.1)	46.5 (10.0)	0.97
TG (mg/dL)	118.6 (55.2)	136.1 (73.2)	0.05	166.7 (103.1)	170.7 (114.0)	0.63
Group Classification (*n*)						
Reduced risk factors	-	18		-	43	
Consistent risk factors	-	14		-	16	
Increased risk factors	-	14		-	1	

MetS, metabolic syndrome; SD, standard deviation; BMI, body mass index; WC, waist circumference; SBP, systolic blood pressure; DBP, diastolic blood pressure; FBS, fasting blood sugar; HDL-C, high-density lipoprotein cholesterol; TG, triglyceride.

**Table 2:** Engagement differences between the groups.

Variables	Pre-MetS			MetS		
	Prevention (*n* = 32)	Non-Prevention (*n* = 14)	*p*-Value	Improvement (*n* = 43)	Non-Improvement (*n* = 17)	*p*-Value
The frequency of healthcare device after 14 weeks, mean (SD) per week						
Weight Scale	3.8 (2.8)	3.7 (2.3)	0.47	3.2 (2.7)	2.6 (2.5)	<0.01
Smart tape measure	3.4 (2.9)	2.7 (2.5)	<0.01	3.1 (2.7)	2.6 (2.7)	<0.05
Blood pressure monitor	3.4 (2.8)	3.1 (2.4)	0.14	3.1 (2.6)	2.2 (2.5)	<0.01
SMBG	2.3 (2.1)	1.8 (1.7)	<0.01	1.9 (1.9)	1.4 (1.6)	<0.01
Smartwatch	5.3 (2.8)	4.4 (3.2)	<0.01	5.2 (2.9)	4.2 (3.1)	<0.01
Total number of use days	18.3 (10.1)	15.7 (9.2)	<0.01	16.5 (9.7)	13.0 (9.6)	<0.01
Physical activity	7162.4 (4714.0)	5837.1 (5034.0)	<0.01	7444.3 (5337.3)	4878.6 (4543.1)	<0.01
Criteria for achievement, mean (SD) per 26 weeks						
Engagement	10.6 (6.1)	9.9 (6.3)	0.71	10.2 (5.8)	7.1 (6.1)	0.07
Persistence	8.9 (5.7)	6.8 (4.7)	0.23	7.5 (4.8)	5.4 (4.9)	0.13
Change of risk factors (*n*, %)						
Baseline *	1.6 (0.5)	1.7 (0.5)	0.56	3.7 (0.7)	3.3 (0.5)	<0.05
WC	14 (43.8)	6 (42.9)		41 (95.3)	13 (76.5)	
Blood pressure	30 (93.8)	10 (71.4)		38 (88.4)	15 (88.2)	
FBS	3 (9.4)	2 (14.3)		26 (60.5)	12 (70.6)	
HDL-C	2 (6.3)	3 (21.4)		27 (62.8)	7 (41.2)	
TG	3 (9.4)	3 (21.4)		25 (58.1)	9 (52.9)	
Follow-up *	1.0 (1.0)	2.92 (0.3)	<0.01	2.0 (1.0)	3.4 (0.5)	<0.01
WC	8 (25.00)	8 (57.1)		22 (51.2)	13 (76.5)	
Blood pressure	13 (40.62)	10 (71.4)		17 (39.5)	16 (94.1)	
FBS	2 (6.25)	4 (28.6)		9 (20.9)	9 (52.9)	
HDL-C	3 (9.37)	10 (71.4)		19 (44.2)	9 (52.9)	
TG	5 (15.6)	9 (64.3)		17 (39.5)	10 (58.8)	

* Baseline and follow-up are expressed as the mean (SD) of number of risk factors. MetS, metabolic syndrome; SD, standard deviation; SMBG, self-monitoring blood glucose; WC, waist circumference; FBS, fasting blood sugar; HDL-C, high-density lipoprotein cholesterol; TG, triglyceride.

## Data Availability

Not applicable.
